# Small Bowel Carcinomas Associated with Immune-Mediated Intestinal Disorders: The Current Knowledge

**DOI:** 10.3390/cancers11010031

**Published:** 2018-12-29

**Authors:** Paolo Giuffrida, Alessandro Vanoli, Giovanni Arpa, Arturo Bonometti, Ombretta Luinetti, Enrico Solcia, Gino Roberto Corazza, Marco Paulli, Antonio Di Sabatino

**Affiliations:** 1 First Department of Internal Medicine, University of Pavia and Fondazione IRCCS Policlinico San Matteo, 27100 Pavia, Italy; paolo.giuffrida01@universitadipavia.it (P.G.); gr.corazza@smatteo.pv.it (G.R.C); 2 Anatomic Pathology Unit, Department of Molecular Medicine, University of Pavia and Fondazione IRCCS Policlinico San Matteo, 27100 Pavia, Italy; alessandro.vanoli@unipv.it (A.V.); giovanni.arpa90@gmail.com (G.A.); arturo.bonometti11@gmail.com (A.B.); o.luinetti@smatteo.pv.it (O.L.); solciae@smatteo.pv.it (E.S.); m.paulli@smatteo.pv.it (M.P.)

**Keywords:** coeliac disease, Crohn’s disease, dysplasia, histotype, overall survival, tumour-infiltrating lymphocyte

## Abstract

Small bowel carcinomas (SBC) are uncommon neoplasms, whose predisposing conditions include hereditary syndromes and immune-mediated intestinal disorders including coeliac disease (CD) and Crohn’s disease (CrD). Although both CD-associated SBC (CD-SBC) and CrD-associated SBC (CrD-SBC) arise from an inflammatory background, they differ substantially in tumour cell phenotype, frequency of microsatellite instability and nuclear β-catenin expression, as well as in prognosis. For these patients, high tumour-infiltrating lymphocyte density and glandular/medullary histotype represent independent positive prognostic factors. Dysplasia adjacent to SBC is rare and characterized by intestinal phenotype and nuclear β-catenin in CD, while it is frequent and typified by gastro-pancreatobiliary marker expression and preserved membranous β-catenin in CrD. Recent evidence suggests that Epstein-Barr virus-positive dysplasia and SBC, albeit exceptional, do exist and are associated with CrD. In this review, we summarize the novel pathological and molecular insights of clinical and therapeutic interest to guide the care of CD-SBC and CrD-SBC.

## 1. Introduction

Small bowel carcinomas (SBC) are remarkably uncommon neoplasms, mostly sporadic. Notwithstanding, several predisposing conditions such as hereditary syndromes –familial adenomatous polyposis, Lynch syndrome, Peutz-Jeghers syndrome, and juvenile polyposis syndrome- and chronic immune-mediated intestinal disorders—coeliac disease (CD) and Crohn’s disease (CrD)—are well-known (Table 1) [1]. The underlying intestinal disorder, i.e., CD or CrD, has been demonstrated to be a stage-independent prognostic factor in patients undergoing surgery for SBC [2]. Although both CD and CrD are sustained by similar pathogenic mechanisms, namely T helper 1 and 17 immune responses [3], CD-associated SBC (CD-SBC) and CrD-associated SBC (CrD-SBC) represent distinct entities in terms of clinical, histopathological, and molecular features (Table 2). The jejunum is the most frequent location for SBC in CD [2], an immune-mediated enteropathy induced by dietary gluten in genetically susceptible individuals [4]. CD-SBC exhibit a high frequency of microsatellite instability (MSI), increased tumour-infiltrating T lymphocytes (TIL), a glandular histotype, and an intestinal phenotype [2,5,6,7]. On the other hand, SBC often localise in the inflamed ileum in CrD [2,8], a form of relapsing transmural inflammatory bowel disease due to an abnormal immune response to commensal microbiota [9]. In particular, SBC always arise in CrD patients with small bowel involvement [8,10]. Most CrD-SBC are microsatellite stable, have low TILs and often show a non-glandular histotype coupled with a non-intestinal phenotype [2,7,8,10,11,12].

Understanding oncogenic pathways of SBC is a need with translational implications in order to better characterise this neoplasm. We here review the current knowledge of CD-SBC and CrD-SBC by highlighting new molecular insights from clinical perspectives, in particular in predicting outcome and response to novel therapeutic options.

## 2. Epidemiology and Risk Factors

Although small intestine represents the 75% of digestive tract length and the 90% of digestive absorptive surface [28], SBC are relatively rare neoplasms and account for less than 5% of all gastrointestinal cancers [29]. However, they correspond to around 40% of all small bowel malignancies [29]. The estimated incidence of SBC varies between 3250 and 5300 cases per year in the USA [30,31], whereas it is about 3600 annual new cases in Europe [32]. The relative risk of developing SBC in CD and CrD raise 14 and 33 times in comparison to the general population, respectively [33,34]. Amongst all SBC, the 13% and the 7% seem to be associated with CD and CrD, respectively [13,34]. The epidemiological features of SBC differ on the basis of underlying chronic immune-mediated intestinal disorders. The median age at CD-SBC diagnosis has been estimated from 53 to 62 years in American, British, Dutch and Italian patients [2,5,6,13,14], while that at CrD-SBC diagnosis seems to be younger varying from 42 to 53 years in most studies (Table 3) [8,10,11,12,15,16,17,18,19,20,21]. Conversely, in an American large-scale retrospective cohort study, CrD-SBC patients presented at a median age of 72.9 years [22]. Recently, the Small Bowel Cancer Italian Consortium also showed an older median age at CrD-SBC diagnosis, i.e. 59 years [2]. We speculate that this discrepancy might be explained by an older age at CrD diagnosis in the latter cohort (50 years) [2] and by a better clinical management of CrD over the last two decades. Sporadic SBC (spo-SBC) patients generally have a higher median age at diagnosis -between 56.5 and 72.1 years- in comparison to both CD-SBC and CrD-SBC [2,5,10,17,20,22]. Risk factors for CD-SBC and CrD-SBC have not thoroughly investigated. According to the well-established protective effects of adherence to gluten-free diet against neoplastic complications in CD [13,35], we believe that a strict gluten-free diet could also reduce the risk of CD-SBC development. Accordingly, Elfström and colleagues [36] demonstrated that the risk of small bowel malignancies in coeliac patients decreases, though not disappearing, after the first year of follow-up, likely by reducing intestinal inflammation and mucosal damage [37].

However, several CD-SBC have been reported in patients under gluten-free diet; therefore, other factors have to be implicated. Interestingly, the median age at CD diagnosis in patients, who develop SBC, ranges from 49 to 59 years (Table 3) [2,5,6], around two-three decades higher than that of patients with CD not evolving into malignant complications [38]. Thus, the diagnostic delay, a known risk factor for refractory CD and, consequently, for enteropathy-associated T-cell lymphoma [39], has been supposed to play a role in CD-SBC. However, only one SBC case has been hitherto described in association with refractory CD [2], a finding suggesting a different pathogenesis between SBC and enteropathy-associated T-cell lymphoma in CD. Conversely, there is no evidence for a role of diagnostic delay in CrD-SBC development in most studies (Table 3), although diagnostic delay is frequently associated with complicated CrD behavior, such as stricturing and penetrating phenotypes [40]. Risk factors reported for CrD-SBC include a long disease duration, a small bowel involvement, a stricturing phenotype and bypassed segment(s) of small bowel [41]. As regards long disease duration, in a French study involving 1935 patients affected by CrD with small bowel involvement at diagnosis a cumulative risk of SBC has been assessed as 0.2% and 2.2% after 10 and 25 years of follow-up, respectively [17]. Although use of 6-mercaptopurine seemed to be a risk factor for CrD-SBC in an American study including seven cases [42], no medical treatment has been unquestionably found to be associated with SBC in larger cohorts [18,43]. On the other hand, small bowel resection and use of salicylates for more than two years protect against SBC in patients with CrD [18]. As regards gender, the rates of female prevalence are extremely heterogeneous in both CD-SBC (25–62%) [2,5,6,13,14] and CrD-SBC (29–60%) [2,8,10,11,12,15,16,17,18,19,20,21,22] so that it is hard to assess a gender predominance in either conditions. However, considering the strong prevalence of CD in women [4], these data may suggest that male gender is at higher risk to develop CD-SBC.

The incidence of SBC cases as a whole is doubled in African Americans (from 10.2 to 14.1 per 1,000,000) compared to Caucasians (from 4.5 to 7.2 per 1,000,000) [44,45]. Conversely, CrD-SBC have been demonstrated to affect more frequently Caucasians in an American large-scale retrospective study from 1992 to 2010 [22]. Likewise, CD-SBC have been reported exclusively in Caucasians in the only study analyzing ethnic differences in this clinical subgroup [13]. It appears that more extensively investigation of epidemiology and risk factors are needed.

## 3. Histopathology and Molecular Biology

In general, SBC as a whole show a predominance (52–60%) of glandular-type histology, i.e., adenocarcinoma [7]. Nevertheless, medullary-type cancers have been reported in association with CD-SBC [7,46], whereas poorly cohesive, diffuse-type neoplasms or mixed glandular/diffuse cases are more common in CrD-SBC in comparison to CD-SBC and spo-SBC [7,17]. Most CD-SBC, as well as spo-SBC, express intestinal phenotype markers, i.e. the caudal-related homeobox transcription factor (CDX)2, the goblet cell marker mucin (MUC)2, cytokeratin (CK)20 and/or the small bowel brush border marker CD10, while CrD-SBC often feature a metaplastic gastro-pancreatobiliary phenotype, characterized by positivity for the gastric foveolar marker MUC5AC and/or the pancreatobiliary duct marker CK7 [7,20].

A high density of CD3^+^ and CD8^+^ TILs mark CD-SBC, while TILs are generally low in CrD-SBC and spo-SBC [2]. This finding points to a greater host immune response against tumour in CD-SBC in comparison with CrD-SBC and spo-SBC, thus leading to a better clinical outcome described in CD-SBC (Table 3). However, this does not prevent tumour growth, probably due to an increased immune tolerance. In particular, it has been hypothesized that programmed cell death ligand 1 (PD-L1) and programmed cell death protein-1 (PD-1), crucial immune checkpoints aimed at inhibiting and escaping immune surveillance, are also implicated in non-familial SBC as well as in colorectal and gastric cancers with MSI and/or Epstein-Barr Virus (EBV) infection [47,48]. An American study on 42 spo-SBC demonstrated PD-1 expression on intratumoural and peritumoural lymphocytes in most cases, and PD-L1 expression on neoplastic cells and immune cells, mainly histiocytes, in a minority of cases [49]. Our preliminary data showed an association between PD-L1 immunoreactivity and TIL density/MSI status in a series of non-familial SBC, including CD-SBC and CrD-SBC [unpublished data]. To the best of our knowledge, no study investigated clonality of TILs in CD-SBC.

Table 4 shows the studies investigating molecular alteration in the cohorts including at least 5 cases of CD-SBC and/or CrD-SBC. MSI, which is a consequence of defective DNA mismatch repair and is verified by mean of molecular and/or immunohistochemical analysis, is found in around one third of all non-familial SBC with significant differences between CD-SBC (65–73% MSI) [2,5,6], CrD-SBC (0–16% MSI) [2,8,10,11,12], and spo-SBC (9–35%) [2,5,10,23,24] (Table 2 and Table 4). MSI induces the anti-tumour immune response supposed to play a crucial role in sustaining a more favourable outcome in these tumours. Genomic profiling of spo-SBC revealed a series of genetic alteration affecting most commonly *TP53* (mutated 58% of cases), *KRAS* (53.6%), *APC* (26.8%), *SMAD4* (17.4%) and *PIK3CA* (16%) [50,51]. As regards CD-SBC and CrD-SBC, overexpression of the *TP53* gene product has been found in about half of cases in both groups, thus confirming the key role of *TP53* alterations in small bowel carcinogenesis [2,8,10], as well as in inflammatory bowel disease-associated colorectal cancers [52]. *KRAS* mutation, which represents an early change in the adenoma–carcinoma sequence of colorectal cancer, has been also found in 31% of CD-SBC and in 12–43% of CrD-SBC [2,8,10,12,53].

Promoter hypermethylation of *APC* has been demonstrated in 73% of CD-SBC, whereas nonsense *APC* mutations have not been reported in CD-SBC [6]. Likewise, allelic loss of *APC* gene was rare in CrD-SBC [10]. However, the involvement of Wnt/β-catenin pathway has been observed in most CD-SBC and spo-SBC, as suggested by aberrant nuclear expression of β-catenin [7,54,55]. On the contrary, nuclear translocation of β-catenin has been rarely identified in CrD-SBC [7,8]. To the best of our knowledge, there are no data on *SMAD4* mutation frequency in CD-SBC and CrD-SBC. *BRAF* V600E mutation, which is extremely rare in spo-SBC [54], is also absent in both CD-SBC and CrD-SBC [2,10,11] or detected up to 7% of CrD-SBC in other studies [8,12]. Therefore, unlike colorectal cancer [56,57], *BRAF* mutation does not seem to play a role in inducing *MLH1* gene methylation, a frequent finding in CD-SBC [2,6]. No significant difference has been observed in *PIK3CA* or *NRAS* mutation rate amongst CD-SBC, CrD-SBC and spo-SBC [2,8]. In addition, genomic profiling identified potentially targetable genetic alterations in most SBC cases (91%) [50]. Laforest and colleagues [58] found *ERBB2*/*HER2* alterations in 12% of spo-SBC, through mutations (7 cases) or amplifications (3 cases). Our group first described *HER2* amplification in two CD-SBC and in two CrD-SBC [2].

## 4. Pathogenesis and Preneoplastic Lesions

The pathogenesis of non-familial SBC is largely unknown due to their rarity. Although dysplastic lesions adjacent to CD-SBC are remarkably rare [7,14,59], the recurrent presence of dysplasia in the superficial part of CrD-SBC as well as in that of spo-SBC has been identified [7,8,60,61]. In particular, in both CrD-SBC and CD-SBC, dysplasia has been described as either flat or raised [7,8,10,11,12,59]. Dysplasia is distant or adjacent to CrD-SBC [7,8,10,11,12], while no dysplasia distant from SBC has been reported in coeliac patients [7,59].

**Figure 1:**
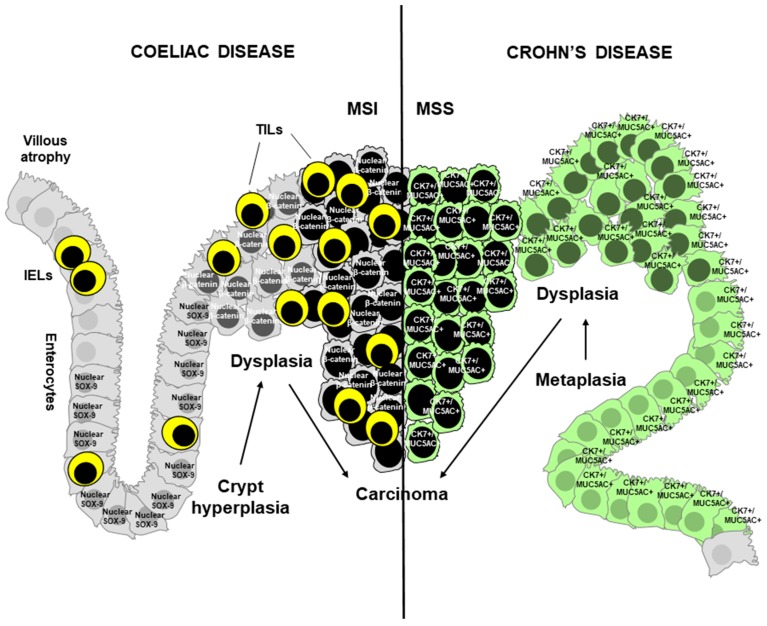
Schematic representation of the pathogenic mechanisms underlying small bowel carcinomas associated with chronic intestinal disorders. In coeliac disease villous atrophy induces crypt hyperplasia, characterised by increased intraepithelial lymphocytes (IEL) similarly to atrophic epithelium. Nuclear Sex-determining Region Y-Box (SOX)-9-positive immature hyperplastic crypts evolve into flat nuclear β-catenin-positive dysplasia, thus leading to coeliac disease-associated carcinoma (CD-SBC). CD-SBC is associated with microsatellite instability (MSI) and high number of tumour-infiltrating lymphocytes (TIL). In Crohn’s disease gastric (MUC5AC^+^)/pancreatobiliary (CK7^+^) metaplasia evolves into dysplastic polypoid growth, which lastly becomes Crohn’s disease-associated carcinoma (CrD-SBC). CrD-SBC is almost always microsatellite stable (MSS).

Overexpression of p53 and retained reactivity for mismatch repair proteins are typical features of dysplasia adjacent to both CD-SBC and CrD-SBC [7]. Of note, loss of MLH1 is rarely identified in dysplasia associated with MSI CD-SBC [7], thus suggesting that *MLH1*-hypermethylation-related MSI is a late process during CD-SBC carcinogenesis. Moreover, the rare dysplastic foci close to the invasive CD-SBC have been found to express nuclear β-catenin, to be compared to the preserved membranous expression of β-catenin in CrD dysplasia [7]. Thus, Wnt pathway activation seems to be an early event in small bowel carcinogenesis in coeliac patients. In keeping with that, overexpression of the Wnt-related transcription factor and stem cell marker Sex-determining Region Y-Box (SOX) 9 has been observed in hyperplastic crypts of untreated CD patients [62], as well as in CD-SBC tumour cells, in direct continuity with SOX-9^+^ adjacent dysplastic and hyperplastic crypts (Figure 1) [7]. This finding may suggest a histogenetic link between crypt hyperplasia and CD-SBC. On the contrary, gastropancreatobiliary metaplastic changes have been predominantly observed in dysplastic or non-dysplastic mucosa close to CrD-SBC [7,20]. Although small bowel dysplasia has been reported to have a low sensitivity (33%) at enteroscopy in CrD patients at high risk of SBC [63], the identification of MUC5AC-positive or CK7-positive metaplastic changes at perendoscopic biopsies should lead CrD patients to a strict endoscopic follow-up. Immature crypt hyperplasia and gastropancreatobiliary metaplasia might be regarded as putative preneoplastic lesions, likely able to evolve into dysplasia and carcinoma, in CD and CrD, respectively (Figure 1). Therefore, we speculate that an inflammation-hyperplasia-dysplasia-carcinoma sequence occurs in CD-SBC development, whereas an inflammation-metaplasia-dysplasia-carcinoma sequence takes place in CrD-SBC pathogenesis. Additional extensive and prospective studies are necessary to confirm such models of cancerogenesis in order to recognise initial preneoplastic lesions, which may aid in early cancer diagnosis.

Lytic phase of EBV infection is a frequent event in inflammatory bowel disease, especially in patients who have overused immunomodulators, in particular corticosteroids [64]. Recently, latent phase of EBV infection, known to have a crucial role in gastroesophageal EBV carcinogenesis [65], has been documented in two microsatellite-stable T-cell rich CrD-SBC [25,26]. In both cases EBV has been also found in dysplastic lesions associated with CrD-SBC and in small foci of iuxta-tumoural epithelium apparently in the absence of dysplasia [25,26]. On this basis we support that rarely EBV latent infection might be a very early, crucial event during carcinogenesis in those patients. Up-to-now, no latent infection with EBV has been found in CD-SBC, while EBV does not seem to be involved in the pathogenesis of spo-SBC, as supported by its absence in a cohort of 56 spo-SBC [27].

## 5. Clinical Presentation and Diagnosis

Duration of the underlying immune-mediated disorder prior to SBC diagnosis differs (Table 3). CD-SBC presented after a median of 1.4–17 years from CD diagnosis comparing to 7–25.2 years from CrD diagnosis in CrD-SBC in studies with the most numerous cohorts of patients with CD-SBC and CrD-SBC, respectively [2,6,8,10,13,14,15,16,17,18,19,20,21]. Notwithstanding, SBC might be diagnosed in a few cases at the same time of underlying inflammatory disorder for both CD and CrD [2,6,10,11,14,17,18,21]. The clinical spectrum of SBC at onset is wide and related to bleeding with subsequent anaemia, positive fecal occult blood test, melena or coffee ground vomiting, to obstruction with symptoms of nausea, vomiting, abdominal pain and unexplained weight loss, or to intussusception and perforation in the locally advanced lesions [1].

**Figure 2:**
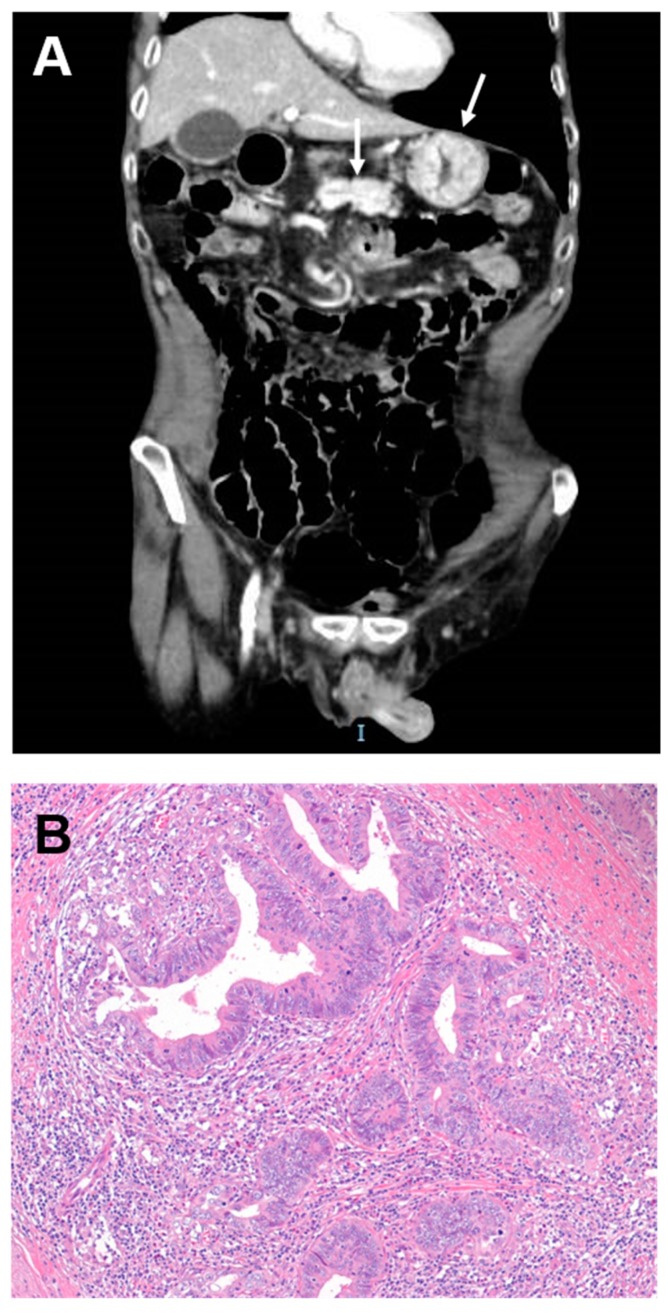
(**A**,**B**) Radiologic and histologic images of a coeliac disease-associated small bowel carcinoma. (**A**) Computed tomography shows a circumferential mass with shouldered borders causing the wall thickening in the duodenum (arrows). (**B**) Haematoxylin and eosin staining shows a glandular-type carcinoma with a high tumour-infiltrating lymphocyte density. Original magnification: 100×.

In patients with a known diagnosis of CD any of the aforementioned symptoms apart from an isolated anaemia should raise the suspicion for SBC. When concomitant diarrhoea and fever are present, first of all enteropathy-associated T-cell lymphoma has to be ruled out [39], Similarly, in the presence of diarrhoea and intestinal obstruction, ulcerative jejuno-ileitis has to be considered [4]. Once CD-SBC is suspected, an upper endoscopy is recommended in coeliac patients in order to find and sample the lesion, if it is located proximally to the ligament of Treitz (Figure 2). However, as most CD-SBC are jejunal, further diagnostic tests, such as device-assisted enteroscopy, computed tomography enterography and magnetic resonance enterography, are usually needed [66]. Conversely, capsule endoscopy should not be indicated in symptomatic patients with SBC due to its several limitations, including the impossibility to take biopsies for histologic diagnosis and the risk of capsule retention and of missing SBC, in particular in case of proximal site.

In patients with a known diagnosis of CrD, obstruction is more likely expected to be the manifestation of fibrostenosing behaviour [67]. Likewise, anaemia and positive fecal occult blood test are generally related to active phases of CrD [68]. Therefore, apart from acute bleeding, all the other symptoms of SBC are hard to discriminate from a relapse of CrD [69]. This accounts for the fact that most diagnoses of CrD-SBC are made during the surgery or even post-operatively by the pathologist [70]. Failure to respond to anti-inflammatory therapies could not be considered *per se* an indicator of CrD-SBC, as it frequently occurs in CrD with intestinal strictures without an inflammatory component [67]. On the other hand, the onset of obstructive symptoms and anaemia in a patient with longstanding quiescent CrD should raise the suspicion for SBC [19]. Ileocolonoscopy is a diagnostic tool only in CrD-SBC located in the last tract of terminal ileum. Otherwise, as nearly all CrD-SBC are located more proximally, retrograde per anal device-assisted enteroscopy is the best tool to identify and biopsy CrD-SBC. Computed tomography enterography and magnetic resonance enterography might help in finding the correct SBC site prior to enteroscopy or laparoscopic surgery [21]. However, computed tomography enterography and magnetic resonance enterography are highly suggestive of CrD-SBC only in a minority of cases showing small bowel mass with localized lymphadenopathy and/or evidence of distant spread, such as liver metastasis or peritoneal carcinomatosis [10,21]. However, the assessment of imaging data by a radiologist specialized in gastrointestinal system could improve the identification of CrD-SBC-related features, including annular mass, nodularity at the extraluminal margins of mass and perforation [21].

In the absence of familial adenomatous polyposis, Lynch syndrome, Peutz-Jeghers syndrome and juvenile polyposis syndrome, both CD and CrD should be considered in any patient with SBC diagnosis.

## 6. Prognosis and Treatment

SBC prognosis is generally poorer than that of colorectal carcinoma [71]. This finding seems true even in CrD patients, in whom SBC have been found to be more aggressive than large bowel cancers [15]. In an American retrospective study, which enrolled 491 SBC mostly spo-SBC, but also CD-SBC (*n* = 13), CrD-SBC (*n* = 23) and SBC associated with familial adenomatous polyposis (*n* = 10), the median overall survival time was 20.1 months, with a 5-year overall survival rate of 26% [72]. The primary reason for this bad outcome is that patients are often asymptomatic until late disease, and metastases are often already present at SBC diagnosis. Tumour stage has been considered the single most important prognostic factor in all SBC [72]. Poor prognosis is also associated with further factors, such as poor differentiation, positive margins, duodenal site, male gender, black ethnicity and older age at SBC diagnosis [31]. High positive lymph nodes-to-total lymph node ratio and a low number of assessed lymph nodes have been correlated with a reduced survival [72,73,74].

However, overall survival significantly differs between patients with CD-SBC and those with CrD-SBC (Table 3). In particular, the predisposing chronic immune-mediated intestinal disorder, i.e., CD or CrD, has been demonstrated to be a stage-independent prognostic factor in patients undergoing surgery for SBC in the largest study systematically comparing CD-SBC, CrD-SBC and spo-SBC [2]. Five-year overall survival rate is relatively high in CD-SBC, i.e., 64.2% and 83% in an American study and in an Italian study recruiting 17 and 26 patients, respectively [2,5]. Conversely, five-year overall survival rate appears to be worse in CrD-SBC patients, ranging from 26% to 38%, in French, Danish and Italian studies [2,12,17]. In keeping with that, two-year overall survival in CrD-SBC has been reported to be 52% and 56% in an American study and in a French study, respectively [10,21], even lower than five-year overall survival in CD-SBC. Overall survival has been found to be more favourable in CD-SBC in comparison with spo-SBC [2,5], whereas no survival difference has been demonstrated between CrD-SBC and spo-SBC [2,10,17,22]. However, in the large study by Wieghard and colleagues [22] patients with CrD-SBC were diagnosed at an earlier stage (I/II) compared with spo-SBC (55% vs. 32%, *p* < 0.0001) and were more likely to undergo surgery (81% vs. 72%, *p* = 0.0016).

Independently of the clinical group, CD or CrD, prognostic factors for SBC include stage, tumour histotype and high TIL density [2,7]. Tumour histology by itself is clinically relevant, as it has been shown how diffuse-, mixed- and solid-types cumulatively considered tend to have a worse prognosis compared to glandular-type and medullary-type cancers [7,46]. Amongst prognostic factors within the CD-SBC group, both MSI and high TILs have been also identified and they correlate one each other [2]. However, only TIL density retains a prognostic power in a multivariable model, presumably due to the fact that several high-TIL SBC showing a good prognosis miss MSI [2]. High TILs in SBC can be induced by additional factors besides MSI status, such as oncogenic viruses. As these regards, it is interesting to note that two non-MSI high-TIL SBC with EBV latent infection described in CrD seem to be associated with a favourable outcome [25,26], probably due to the anti-tumour immune response induced by abnormal peptide production from EBV. Therefore, although these findings need to be confirmed by further evidence, EBV latent infection should be considered in CrD-SBC for a better prognostic evaluation.

Currently, therapies for CD-SBC and CrD-SBC largely derive from recommendations for spo-SBC [75]. Surgery is the mainstay of curative treatment for SBC without distant metastasis (M0), whose possible benefits from adjuvant chemotherapy are controversial [1]. Surgical resection with suitable lymph node sampling is necessary for long-term survival in resectable SBC. Surgery is the only curative therapy for SBC at stage I, whereas it should be followed by adjuvant chemotherapy, such as FOLFOX4 or LV5FU2 or oral fluoropyrimidine for SBC at stage II or III [75]. Systemic chemotherapy is the treatment for non-resectable or metastatic SBC, namely those at stage IV [75]. Several molecular alterations may suggest the response to novel therapies. In particular, *KRAS* wild-type mutational status has been demonstrated to predict responsiveness to anti-epidermal growth factor receptor monoclonal antibodies cetuximab and panitumumab alone or combined with chemotherapy in metastatic SBC in several case reports [76,77]. Conversely, a recent phase 2 clinical trial of panitumumab showed no response rate of this drug in nine patients with metastatic *KRAS* wild-type SBC, of which one was associated to inflammatory bowel disease and two to Lynch syndrome [78]. In particular, in this trial seven patients had a progression of SBC, whereas the remaining two ones showed stable SBC [78]. It has been hypothesized that SBC, as well as right-sided colon carcinomas, benefit less from anti-epidermal growth factor receptor agents than left-sided colon carcinomas due to their distinct embryologic origin, namely midgut for SBC and right-sided colon carcinomas and hindgut for left-sided colon carcinomas [78,79]. Although *HER2* amplification is rare in CD-SBC and CrD-SBC [2], it is worth being investigated as a potential therapeutic target of anti-HER2 receptor monoclonal antibody trastuzumab [58,80]. Expression of PD-L1 on tumoural and immune cells in SBC should encourage clinical trials aimed at assessing efficacy of anti-PD-L1 monoclonal antibodies avelumab and atezolizumab [49]. In that regard, an open-label phase 2 clinical trial of avelumab is underway in advanced and metastatic SBC [81]. Likewise, an open-label phase 2 clinical trial has been investigating the efficacy of atezolizumab in combination with MEK inhibitor cobimetinib in advanced rare neoplasms, including SBC [82]. The use of anti-PD-1 monoclonal antibodies pembrolizumab and nivolumab might be appropriate in a subset of metastatic MSI SBC [83]. Currently, an open-label phase 2 clinical trial of pembrolizumab is ongoing in non-resectable metastatic or locally advanced SBC [84]. In addition, pembrolizumab has been testing in a large phase 1b clinical trial together with the Hsp90 inhibitor XL888, which inhibits Hsp90 chaperone function and promotes the proteasomal degradation of several oncogenic signaling proteins, such as Her-2 and Met [85]. This trial was designed for many advanced gastrointestinal neoplasms, including SBC too, in order to identify the recommended phase 2 dose for the combination of XL888 and pembrolizumab [85]. Another clinical trial has been assessing response to nivolumab plus the anti-CTLA-4 monoclonal antibody ipilimumab combination immunotherapy in patients with advanced rare neoplasms, including SBC [86]. Briefly, immunotherapy has been changing the therapeutic scenario in several solid tumours, in particular PD-1/PD-L1 pathway blockade should be considered in advanced MSI SBC, as mismatch repair deficiency has been demonstrated to predict response to anti-PD-1 antibodies in eleven types of solid tumours, including SBC [87].

## 7. Conclusions and Perspectives

It is well-known that chronic inflammation plays a pivotal role in the carcinogenesis of gastrointestinal tract. Although both CD and CrD are disorders sustained by T helper 1 and 17 immune responses, the outstanding differences between CD-SBC and CrD-SBC are remarkable. However, the pathogenic mechanisms underlying CD and CrD show substantial discrepancies regarding immune cell types and pro-inflammatory cytokines implicated. Up-to-now, no surveillance programme has been identified for this uncommmon neoplastic complication in patients with CD or CrD. CD-SBC patients harbour MSI and high TIL more frequently and show a better outcome than CrD-SBC. This seems mainly due to their higher TIL density, which at multivariable analysis showed an independent prognostic value. The prognostic power of histologic structure was independent of the clinical groups, while the non-intestinal phenotype associated with poor outcome was dominant among CrD-SBC. Immature crypt hyperplasia and gastropancreatobiliary metaplasia appear to be putative preneoplastic lesions, likely able to evolve into dysplasia and carcinoma, in CD and CrD, respectively. However, it remains to be investigated as whether these two potential histogenetic factors can be exploited in clinical studies aiming to diagnose cancer at an early, even preneoplastic, stage. EBV latent infection should be considered in SBC irrespective of their histologic type, in light of the better prognosis of the only two EBV^+^ CrD-SBC cases reported so far [25,26]. Briefly, regardless of clinical groups MSI/TIL status, *KRAS* mutations, *HER2* amplification, PD-L1, and PD-1 expression might help in stratifying patients for targeted anti-cancer therapy in patients with SBC. Although large-scale phase 3 randomized clinical trials are hard to perform due to SBC rarity, novel clinical trial designs and multi-institutional collaborations are crucial to improve management of patients with this orphan cancer.

## Figures and Tables

**Table 1:** Risk factors for small bowel carcinoma.

Risk Factor
**Inherited Tumour Syndromes**
Familial adenomatous polyposis
Peutz-Jeghers syndrome
Hereditary nonpolyposis colon cancer syndrome (Lynch syndrome)
Juvenile polyposis syndrome
MUTYH-associated polyposis
**Other Genetic Disorders**
Cystic fibrosis
**Immune-Mediated Intestinal Disorders**
Coeliac disease
Crohn’s disease
**Other Causes**
Small bowel sporadic adenomatous polyps
Long-standing ileostomy

**Table 2:** Clinical, histopathological and molecular features of small bowel carcinomas according to the subgroup.

Feature	CD-SBC	CrD-SBC	Spo-SBC
Age at diagnosis	53–62 yrs [2,5,6,13,14]	42–73 yrs [2,8,10,11,12,15,16,17,18,19,20,21,22]	56.5–72.1 yrs [2,5,6,10,17,20,22]
Site	Jejunum and duodenum [2,5,6,13,14]	Ileum [2,8,10,11,12,15,16,17,19,20,21,22]	Jejunum and duodenum [2,5,6,10,17,20]
MSI status	65–73% [2,5,6]	0–16% [2,8,10,11,12]	9–35% [2,5,6,10,23,24]
Tumour cell phenotype	Intestinal [7]	Non-intestinal [7,20]	Intestinal [7]
Oncogenic viruses	Unknown	EBV latent infection [25,26]	No association with EBV infection [27]

CD-SBC, small bowel carcinoma associated with coeliac disease; CrD-SBC, small bowel carcinoma associated with Crohn’s disease; EBV, Epstein-Barr Virus; MSI, microsatellite instability; spo-SBC, sporadic small bowel carcinoma; yr, year.

**Table 3:** Studies on small bowel carcinomas associated with coeliac disease or Crohn’s disease.

Authors, Year	Pt	Age at SBC dgn (Median, Range, yrs)	Age at CD or CrD dgn (Median, Range, yrs)	CD or CrD Duration at SBC dgn (Median, Range, yrs)	Stage III/IV (%)	Overall Survival (%)	Main Findings
**Small bowel carcinoma associated with coeliac disease (CD-SBC)**							
Bruno JC et al., 1997 [14]	6	62, 45–75	NA	17, 0–40	NA	NA	No evidence of flat dysplasia was present
Howdle PD et al., 2003 [13]	23	62 *, 47–80	NA	8.2, 0.8–36	NA	NA	CD-SBC account for 13% of all SBC
Potter DD et al., 2004 [5]	17	59.5, 42–78	53, 25–77	NA	8/17 (47)	64.2 at 5 yrs	CD-SBC have a high incidence of mismatch repair deficiency
Diosdado B et al., 2010 [6]	15	61, 47–79	59, 18–79	2.5, 0–32.3	NA	NA	CD-SBC have promoter hypermethylation of the *APC* gene
Vanoli A et al., 2017 [2,7]	26	53, 28–80	49, 7–79	1.4, 0–25	8/26 (31)	83 at 5 yrs	CD-SBC harbour MSI, high TILs and nuclear β-catenin expression frequently and show a better outcome in comparison with CrD-SBC
**Small bowel carcinoma associated with Crohn’s disease (CrD-SBC)**							
Michelassi F et al., 1993 [15]	7	47.7 *, 33–73	24, 11–57	20, 10–30	NA	6 mos (mean)	Survival is worse in CrD-SBC than in colorectal cancer complicating CrD
Rashid A et al., 1997 [11]	8	45.5, 35–71	33.5	NA, 0–30	0/7 (0)	28.5 mos (median)	CrD-SBC have *RAS* and *TP53* mutations
Sigel JE et al., 1999 [16]	8	42, 35–71	35, 23–52	12, 0.6–19	2/8 (25)	NA	Most CrD-SBC have dysplasia adjacent to carcinoma
Palascak-Juif V et al., 2005 [17]	20	47, 33–72	36, 15–54	16, 0–37	11/20 (55)	35 at 5 yrs	Signet-ring cells were found in 7/20 CrD-SBC
Piton G et al., 2008 [18]	29	45, 29–74	34, 13–63	7, 0–52	NA	NA	Small bowel resection and salicylate intake ≥2 yrs protect against CrD-SBC
Widmar M et al., 2011 [19]	29	55.4, 22–81	25, 13–63	25.2, 0.8–51.3	16/29 (55)	NA	Two clinical indicators of SBC were symptoms in longstanding quiescent CrD and obstruction refractory to medical therapy
Svrcek M et al., 2014 [8]	41	47	NA	13.5	19/41 (46)	NA	40/41 CrD-SBC were observed in inflamed mucosal areas. Flat or raised dysplasia was found in 20/41 patients with CrD-SBC
Whitcomb E et al., 2014 [20]	11	47, 42–77	24, 6–33	25, 10–40	NA	NA	10/11 CrD-SBC expressed at least a gastric marker and 8/11 CrD-SBC expressed the pancreatobiliary marker CK7
Weber NK et al., 2015 [21]	34	52.9, 32–74	22.4, 13.0–69.3	22.3, 0–50.6	NA	52 at 2 yrs	Imaging features suggestive for CrD-SBC included annular mass, nodularity at the extraluminal margins of mass, and perforation
Grolleau C et al., 2017 [10]	9	46, 37–67	36, 10–67	15, 0–32	5/9 (56)	56 at 2 yrs	Adjacent dysplasia was present in 9/9 CrD-SBC
Bojesen RD et al., 2017 [12]	23	53, 37–85	NA	NA	NA	26 at 5 yrs	79% of CrD-SBC showed inflammation-dysplasia-carcinoma sequence
Wieghard N et al., 2017 [22]	179	72.9	NA	NA	71/179 (40)	3.9 yrs (median)	CrD-SBC have similar overal survival compared to sporadic SBC
Vanoli A et al., 2017 [2,7]	25	59, 33–84	50, 22–84	13, 0–41	13/25 (52)	38 at 5 yrs	CrD-SBC exhibit a low rate of MSI and TILs CrD-SBC are associated with dysplasia and metaplasia, both showing gastropancreatobiliary phenotype
Vanoli A et al., 2017 [26]	31	59, 33–84	NA	NA	17/31 (55)	NA	EBV^+^ CrD-SBC may occur

CD, coeliac disease; CK, cytokeratin; CrD, Crohn’s disease; dgn: diagnosis; EBV, Epstein-Barr Virus; mo, month; MSI, microsatellite instability; NA, not available; Pt, patient; SBC, small bowel carcinoma; TIL, tumour-infiltrating lymphocyte: yr, year. *, mean.

**Table 4:** Molecular alterations in small bowel carcinomas associated with coeliac disease or Crohn’s disease.

Authors, Year	Pt	MSI Status N (%)	*KRAS* Mutation N (%)	*NRAS* Mutation N (%)	*BRAF* Mutation N (%)	*PIK3CA* Mutation N (%)	*HER2* AmplificationN (%)	p53 Overexpression N (%)	Nuclear β-Catenin Expression N (%)
**Small bowel carcinoma associated with coeliac disease (CD-SBC)**									
Potter DD et al., 2004 [5]	17	8/11 (73)	NA	NA	NA	NA	NA	NA	NA
Diosdado B et al., 2010 [6]	15	6/9 (67)	NA	NA	NA	NA	NA	NA	NA
Vanoli A et al., 2017 [2,7]	26	17/26 (65)	8/26 (31)	1/26 (4)	0/26 (0)	4/26 (15)	2/26 (8)	12/26 (46)	24/26 (92)
**Small bowel carcinoma associated with Crohn’s disease (CrD-SBC)**									
Rashid A et al., 1997 [11]	8	1/7 (14)	3/7 (43)	NA	NA	NA	NA	4/7 (57)	NA
Svrcek M et al., 2014 [8]	41	1/36 (3)	7/30 (23)	NA	1/29 (4)	0/23 (0)	NA	21/35 (60)	16/31 (52)
Grolleau C et al., 2017 [10]	9	1/9 (11)	1/8 (12.5)	NA	0/8 (0)	NA	NA	NA	NA
Bojesen RD et al., 2017 [12]	23	0/14 (0)	2/14 (14)	NA	1/14 (7)	NA	NA	NA	NA
Vanoli A et al., 2017 [2,7]	25	4/25 (16)	4/25 (12)	1/25 (4)	0/25 (0)	2/25 (8)	2/25 (8)	12/25 (48)	6/24 (25)

MSI, microsatellite instability; NA, not available; Pt, patient.
